# The Legislature, the Governor and the Asylums—Superintendent Wilson Resigns

**Published:** 1899-10

**Authors:** 


THE LEGISLATURE, THE GOVERNOR AND THE ASYLUMS—SUPERINTENDENT WILSON RESIGNS.—It will be remembered that the last Legislature passed an act which requires the superintendents of all the eleemosynary institutions of the State to board themselves and families, and makes them responsible for any possible stealage of provisions by the inmates or employes; requiring superintendents to make quarterly reports of what disposition has been made of said provisions; how much has been eaten, by whom, etc. And it is added that "if the Comptroller shall learn from said report, or from any other reliable source that any provisions have been 'misappropriated,' he shall stop the salary of the superintendent till he pays for the shortage," or words to that effect. In short, the superintendent is to be treated as a thief, and must be watched. The JOURNAL expected to see every superintendent promptly resign; but they didn't.

Had they done so the Legislature, which will meet in called session this winter, would have hastened to undo the very unwise thing, for, we venture the statement that were they to resign, no capable men could be found to take their places. The salary is only $2000 a year. Heretofore the State furnished board and quarters and laundry for the superintendents and their families. This was equivalent to a fairly decent salary. Now, the salary is reduced by the new law, as one of the superintendents told me, to about $800. Of course, as long as capable men will hold such positions at such price, swallowing the affront contained in the bill, the Legislature will not change the law. We are especially gratified to state that Dr. J. T. Wilson, late President of the State Medical Association, one of the best asylum men in the U. S., and who was invited to take the Terrell asylum (didn't apply for it), has resigned, and so far no one has been found to take his place. The appointment has been offered to Dr. John Preston, of Lockhart (and declined), another distinguished asylum man and formerly superintendent of the Terrell asylum, and previously at the Austin asylum, and perhaps to others. In my correspondence with certain distinguished Texas physicians recently, who were once applicants for such appointment, I have been informed that, as one expressed it, they "wouldn't touch it with a forty foot pole." The Governor will experience some difficulty, I think, in getting a competent alienist to take Dr. Wilson's place. Shame on the niggardly policy of asking distinguished gentlemen to serve the State in those difficult positions, positions which require special knowledge and extraordinary qualifications as to administration, and then treating them like pick-pockets, and paying them laborer's wages.

